# Individual product determination in the new Dutch DBC system: how to make the system transparent for its users

**DOI:** 10.1186/1472-6963-11-S1-A17

**Published:** 2011-10-19

**Authors:** R Swiers, R Kleijwegt

**Affiliations:** 1 DBC-Onderhoud, Utrecht, 3526 KS, The Netherlands; 2 Casemix BV, Arnhem, 6845 BH, The Netherlands

## Introduction

On January 1st, 2012, the Dutch declaration system (diagnosis treatment combination), which was introduced in 2005, will be converted. The initial development of this conversion was begun in 2007/2008. In all phases of the conversion, Information technology has supported and will continue to support the system (that is, in early development, in transition, and from 2012 on into production). In our presentation, we would like to detail information regarding the development of this new system and the support given its development by information technology.

## Methods

The initial developments that were part of the new DBC (in English: “Diagnosis Treatment Combination”) system were done in 2007. These developments were the result of an agreement between all the authorities involved in the Dutch system: hospitals, insurance companies, and the government. The development was called ‘Project DOT’, which can be roughly translated as ‘DBC’s On Their Way to Transparency’. DOT was to be developed based on ICD-10 chapters and registration in the current DBC system.

The new system would incorporate three large and interdependent changes:

1. A new basic model, RSAD (in English: Register, Extract, Deduce and Declare).

2. A new product structure to determine products.

3. New rules of registration to determine when to declare.

For the second part – the product structure – DBC-Onderhoud used the ICD-10 chapters and registration available in the current system from the DBC Information System (DIS) to develop decision trees. These trees determine the products for a single period of patient care. This new product structure consists of a number of relevant elements.

The next step was to open the discussion to specialists, and to decide whether these products were logical, and whether they should be expanded or cut in the first drafts. To enable a discussion between different parties, DBC-Onderhoud needed an online tool that was able to make the product structure transparent. This tool, called the “care product viewer”, was built in cooperation with Casemix, a Dutch consultancy organization. It allowed everyone, literally, to see what was built, what had been changed, and what needed to be changed.

## Results

The discussions and iterations on the first draft resulted in the creation of a product structure for inpatient and outpatient hospital care which included approximately 4400 singular products in 123 product groups. These product groups consist of decision trees. Within decision trees, conditions are checked (for example, a registered diagnosis code, or a performed certain-care activity). In the product structure, there are almost 2500 current diagnosis codes, and more than 3000 activity codes, that are used.

This structure can be roughly divided into three categories:

1. Intensive, surgical products.

2. Conservative inpatient care products (diagnostics and small treatment for inpatient care; no large surgery).

3. Conservative outpatient care products (diagnostics and small treatment for outpatient care; no large surgery).

A patient is treated in the hospital and all provided care is registered (such as diagnosis, diagnostics, outpatient consultation, surgeries, patient days, and so on). After a given period, decided by automatic registration rules, the patient dataset is sent to a central web application, the grouper. The grouper supports the rules of the product structure and determines a care product. For many people this feels like being in a black box, and they want to see how a determination works to be able to understand it.

As a result, an online tool was created to allow everyone who was interested to see how a product was determined. The tool shows possible products that can be declared and which type of care – at minimum – should be provided to be able to declare a product. In addition, a wide variety of underlying information about products and product groups is made available.

Because of the possibilities of this tool, today (in 2011) it is being used by hospitals, consultants, hospital representatives, and insurance companies to analyze the impact on their production of the transition to the new system. As well, the tool will allow hospitals to analyze their production from 2012 onward and evaluate whether a faulty or unexpected product has been determined.

The initial product structure will be ready for implementation in 2012. However, we know that additional changes will be needed, and that these will lead to a newer version of the product and changes to its structure. As well, all parties involved will need to be informed of these changes in a timely and accurate manner, so that they can assess them. In addition, transparency will be needed regarding the changes. DBC-Onderhoud has provided that via the online tool.

## Conclusions

When the new system goes ‘live’ in 2012, we will provide Dutch hospitals with a system built from their own registrations, and approved by all representatives involved in Dutch healthcare. This was possible because representatives were able collectively to decide on the development of the system. We will also provide to everyone interested a tool that shows, in great detail, the products and their underlying structural information. ICT has supported the development of this system, and ITC will continue to support and service the product throughout the transition period and into production.

